# [Retracted] Bmi‑1‑targeting suppresses osteosarcoma aggressiveness through the NF‑κB signaling pathway

**DOI:** 10.3892/mmr.2023.12978

**Published:** 2023-03-13

**Authors:** Jiaguo Liu, Bin Luo, Meng Zhao

Mol Med Rep 16: 7949–7958, 2017; DOI: 10.3892/mmr.2017.7660

Following the publication of this paper, it was drawn to the Editor's attention by a concerned reader that the immunohistochemical data shown in Fig. 1C and F, the cell invasion assay data shown in Fig. 2C and D, and all the data shown in Fig. 4D-F were strikingly similar to data appearing in different form in other articles by different authors at different research institutes; moreover, some of the scratch-wound data shown in Fig. 2B appeared to have been duplicated, such that the same data were shown to represent experiments that were meant to have been performed under different experimental conditions. Owing to the fact that the contentious data in the above article were already under consideration for publication prior to its submission to *Molecular Medicine Reports*, the Editor has decided that this paper should be retracted from the Journal. After having been in contact with the authors, they agreed with the decision to retract the paper. The Editor apologizes to the readership for any inconvenience caused.

